# Recent Advancements in Marine Collagen: Exploring New Sources, Processing Approaches, and Nutritional Applications

**DOI:** 10.3390/md23050190

**Published:** 2025-04-28

**Authors:** Joinul Islam, Kevin E. Mis Solval

**Affiliations:** Department of Food Science and Technology, The University of Georgia, Griffin, GA 30223, USA

**Keywords:** marine collagen, gelatin, collagen peptides, collagen extraction, blue proteins

## Abstract

Collagen is a structural protein found in the connective tissues of terrestrial and marine animals. Its diverse functional attributes span its applications in several industries, including food, supplements, cosmetics, and pharmaceuticals. Typically derived from mammalian sources, collagen and its derivatives, including gelatin and collagen peptides, are essential for the food and supplement industries. Recently, marine collagen has emerged as a viable mammalian collagen alternative due to its unique functionality and sustainability. Marine vertebrates and invertebrates are reliable sources of marine collagen. Some marine organisms are promising sustainable sources of collagen for nutritional applications. Recent research highlights significant advances in marine collagen extraction, processing, and novel applications. Hence, recent interest has propelled research in identifying novel collagen sources and advancing technologies to produce marine collagen-based products. Considering the recent scientific interest in marine collagen, this review provides an overview of recent progress in marine collagen production, including novel sources, innovative processing technologies, nutritional and functional properties, safety and quality control, current challenges, and future research directions. The review highlights certain challenges, including unpleasant odor, flavor, color, insufficient supply, and inconsistent quality of marine collagen. Future research should focus on increasing the collagen extraction yield, improving the smell and flavor, and developing novel delivery systems to increase bioavailability and functionality.

## 1. Introduction

Collagen, a complex fibrous structural protein, constitutes approximately 30% of the total protein in many vertebrates and invertebrates [1]. Furthermore, it is an essential extracellular component in various connective tissues, including ligaments, bones, skin, cartilage, and tendons [2]. Interestingly, collagen is one of the most abundant structural proteins found in mammals [3]. Its molecular structure comprises three polypeptide chains forming characteristics of a triple helix with a molecular weight of around 300 kDa. Each polypeptide chain comprises thousands of amino acids, and the most prevalent amino acid sequence is the Gly-X-Y, where proline (Pro) and hydroxyproline are often found in X and Y positions in the peptide chain [4]. Moreover, the typical dimensions of a collagen fibril are about 14–15 Å in diameter and 2800 Å in length [2].

Collagen’s unique properties make it valuable in several food, beverage, cosmetic, and pharmaceutical applications. For example, dietary collagen supplements may support bone, hair, and skin health; meanwhile, collagen-derived products such as gelatin and collagen peptides are promising anti-aging materials with rejuvenating properties; however, the extent of these benefits varies among individuals [5]. Traditional and ancient foods, including bone broth, organ meats, and tendons, are rich in collagen. In contrast, modern diets, including plant-based diets and the reduced consumption of collagen-rich foods, may lead to collagen deficiencies, affecting joint flexibility and skin health, and exacerbating some aged-related conditions in modern consumers [6]. Moreover, collagen synthesis in the human body decreases with age, potentially affecting skin and bone health. The clinical evidence of the health benefits of dietary collagen is still limited; however, preliminary results are promising [7]. Beyond their nutritional benefits, some collagen-derived products (gelatin and collagen peptides) exhibit functional properties that interest the food and beverage industry, such as texturizing, thickening, and gel formation. In addition, they show surface behavior properties, including foam formation, stabilization, emulsification, adhesion, cohesion, and film-forming capabilities [8]. Collagen’s ability to act as a surface-active agent, interacting with lipid-free surfaces, is also notable [9]. These diverse properties have driven the demand for collagen in the food industry [10].

Commercially available collagen-based products are primarily derived from mammalian sauces such as bovine and porcine skin, bone, and tendons due to their high collagen content and well-established extraction techniques [11]. Bovine and porcine-sourced collagens are rich in type I collagen and have long been used in food, pharmaceuticals, and biomedical applications. However, concerns over cost, potential immune response, the risk of disease transmission, and religious restrictions have limited the use of mammalian-sourced collagen [1]. For instance, the recent incidents of diseases like bovine spongiform encephalopathy (BSE), foot-and-mouth disease (FMD), and transmissible spongiform encephalopathy (TSE) have hindered the use of mammalian sourced collagen [12]. In addition, approximately 3% of the global population exhibits allergic reactions to bovine collagen, and cultural groups (Muslims, Hindus, and Jews), who make up 38.4% of the global population, do not consume either porcine or bovine-sourced collagen due to their religious constraints [8]. Hence, these limitations have increased the demand for alternative collagen sources, including those derived from marine organisms. Aquatic species, including fish, jellyfish, sponges, squid, cuttlefish, and octopus, are emerging as viable and sustainable sources of marine collagen [1]. Marine collagen offers unique advantages over mammalian-sourced collagen, including lower immunogenicity, the absence of zoonotic disease risk, and broader acceptability across different cultural and religious groups [8,11]. Furthermore, marine collagens are mainly derived from industrial waste and byproducts, which makes them cost-effective alternatives to mammalian-sourced collagen. According to Srikanya et al. [13], approximately 25% of the total fish weight is utilized in processing industries, which varies based on the processing technology. Notably, fish byproducts, including skin, bone, and scales, may contain nearly 30% collagen, therefore representing a viable source of marine collagen [14].

In 2022, the worldwide collagen market was valued at USD 9.12 billion, and by 2028, it is projected to reach USD 16.6 billion [15]. Specifically, the marine collagen market, benefiting from environmental and religious compatibility, is rapidly growing. Its market value increased from USD 581.3 million in 2017 to USD 1 billion in 2022 and is expected to reach USD 1.91 billion by 2030 [16]. According to Fortune Business Insights [17], the global marine collagen market was valued at USD 1167.3 million in 2024 and is projected to increase from USD 1262.3 million in 2025 to USD 2316.2 million in 2032, reflecting a compound annual growth (CAGR) of 9.06%. The accelerated market expansion of marine collagens suggests a growing demand in different sectors, including food, supplement, cosmetic, and biomedical industries. In the food industry, marine collagen is widely incorporated into functional foods, beverages, and nutritional supplements, due to its high bioavailability, favorable sensory properties, and compatibility with clean-label trends [17]. Beyond its nutritional role as source of protein, marine collagen is also recognized for its potential to deliver essential nutrients such as omega-3 essential fatty acids and bioavailable micronutrients [17]. Thus, tremendous market growth and a growing consumer preference for sustainable sources have spurred scientific interest and research into marine collagen and its derivates. Recently, the number of research articles related to marine collagen and collagen-based products has rapidly increased around the world. Current research efforts are focused on exploring alternative marine collagen sources to satisfy the demands of the food and supplement industries. In addition, this review provides an overview of recent advances in the extraction, processing, and utilization of marine collagen-based products. The nutritional, health, and functional benefits and safety of marine collagens and the current challenges and future research directions are also discussed.

## 2. Chemical Structure and Composition

As previously described, collagen is a structural protein originating in the extracellular matrix of several tissues of vertebrates and invertebrate species, and it is formed by three polypeptide strands (named alpha chains) coiled into a triple-helix structure. Each polypeptide strand is made up of thousands of amino acids and consists of a repeating (Gly-X-Y) amino acid sequence. Notably, Gly is located in each third position, and proline (Pro) and hydroxyproline (Hyp) are predominantly located in the X and Y positions, stabilizing the helix through their unique chemical properties [1]. Furthermore, collagen fibrils are formed by the aggregation of collagen subunits, named tropocollagen (300 nm length and 1.5 to 2 nm diameter), and are the functional units in connective tissues [18]. The triple-helix structure features a right-handed conformation formed by three left-handed polypeptide strands. This structure is stabilized by numerous hydrogen bonds, facilitating the dense packing of the helix [19].

**Figure 1:**
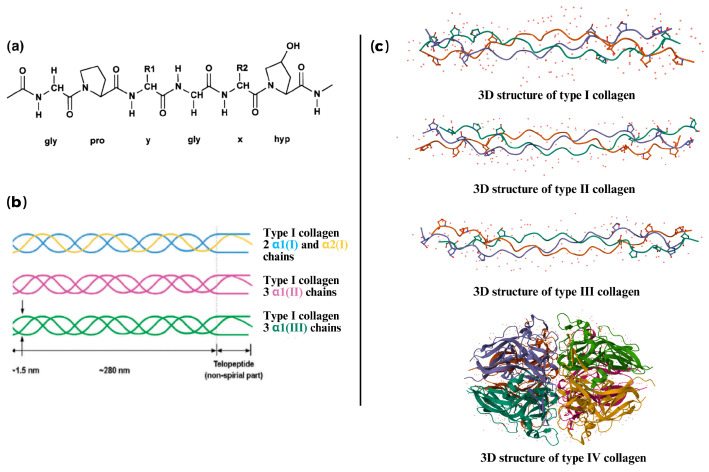
Structure of collagen. (**a**) Primary amino acid sequence of type I collagen, illustrating its characteristic repeating Gly-X-Y, where X and Y are often Pro and Hyp, respectively. (**b**) Schematic representation of collagen molecular assembly, where three polypeptide α-chains are self-assembled to form a right-handed triple helix called tropocollagen and are different in different collagen types: I, II, and III. Type I collagen is composed of two α1(I) chains and one α2(I) chain, Type II collagen consists of three identical α1(II) chains, and Type III collagen consists of three identical α1(III) chains. (**c**) Three-dimensional models of Type I, II, III, and IV collagen molecules. Type I, II, and III collagen form fibrillar networks, while type IV collagen assembles into sheet-like structures and is a major component of basement membranes [9,23,24,25].

There are 29 recognized types of collagen, each distinguished by unique alpha chains and organization structures [1]. Types I, II, and III are the most prevalent, constituting 80–90% of all collagens in organisms, with type I being the most abundant (up to 85% of total collagen) in the human body. According to Skinglo et al. [20], type I collagen is formed by two types of polypeptide α-chains (α1 and α2); meanwhile, type II and type III collagen are three α-chains of the same type. In addition, marine collagen is primarily type I collagen, with smaller quantities of type II. The structure of collagen types I, II, III, and IV is shown in Figure 1. The structure and molecular weight of marine collagen are similar to those of bovine and porcine collagen, with small differences in its amino acid composition [21]. According to Berillis [4], marine collagen can be found in a fibrillar and nonfibrillar state and often shows inferior gelling and melting temperatures and lower viscosities than their mammalian counterparts. This effect may be due to the fact that marine collagens usually have a lower hydroxylysine and total imino acids (proline and hydroxyproline) content compared to mammalian collagens, and higher levels of serine, threonine, and methionine [21]. This variation may influence the marine collagen’s denaturation temperature and functional properties, such as solubility and cross-linking potential. Consequently, marine collagen often shows less favorable gelling properties than mammalian collagen, which limits its food applications. Nonetheless, this limitation can be mitigated by chemical cross-linking methods, such as using 1-ethyl-3-(-3-dimethylaminopropyl) carbodiimide/N-hydroxy succinimide (EDC/NHS) [21]. The amino acid composition of bovine, codfish skin (marine fish), Atlantic salmon, sea bass scale, and jellyfish collagen is illustrated in Table 1. The amino acid composition plays a critical role in determining the functional properties of collagen, including thermal stability, gelling ability, and solubility. For instance, the reduced imino acid content in marine collagen results in lower thermal stability and gelling temperature, making it less suitable for applications that require high heat processing [4,22]. Conversely, marine collagen has higher levels of serine, threonine, and methionine, which may improve its hydrophilicity and solubility. This makes marine collagen suitable for cold-processing applications and functional beverages [21].

## 3. Source of Marine Collagen for Food and Dietary Supplements

Marine collagen, increasingly recognized as a viable mammalian collagen alternative, addresses various health, religious, and environmental concerns [27]. Notably, marine collagens are reported to exhibit higher bioavailability compared to mammalian collagens, potentially due to their lower molecular weight and distinctive amino acid profile—characterized by higher glycine content and lower levels of proline and hydroxyproline [5]. Recently, researchers have focused on utilizing collagen from a variety of marine sources, which can broadly be classified into two main categories: vertebrates and invertebrates.

**Figure 2:**
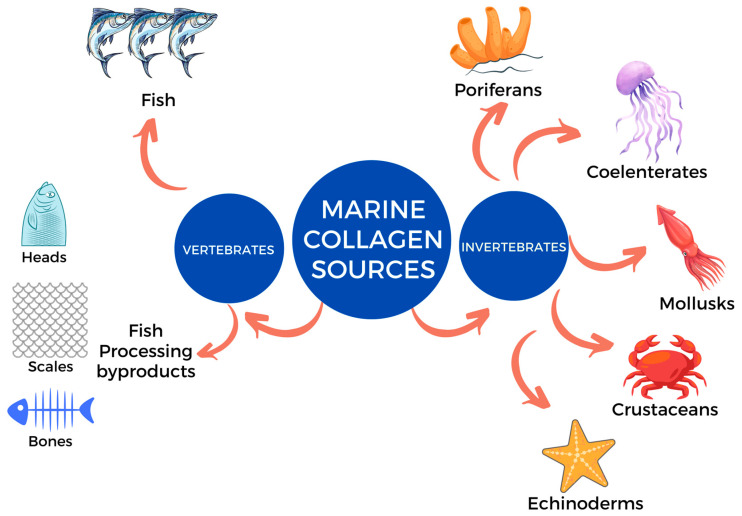
Source of marine collagen.

Marine vertebrates, such as fish, have complex skeletal structures and are typically rich in collagen, especially in their bones, skin, scales, and fins. In contrast, marine invertebrates, which make up most of the marine biodiversity, offer a vast and diverse range of potential collagen sources. Examples of marine invertebrates that contain collagen include poriferans (marine sponges), mollusks (byssus and cephalopod), crustaceans (mantis shrimp), echinoderms (such as starfish and sea urchin), and coelenterates (like jellyfish) [10]. This diversity in marine collagen not only provides a variation in collagen types but also presents unique opportunities for developing novel food applications. Additionally, populations of certain marine invertebrates such as jellyfish, are on the rise, leading to jellyfish blooms or outbreaks. This increase is attributed to factors like climate change, the overfishing of natural predators, and eutrophication. These changes can disrupt fisheries, aquaculture, and tourism [5]. Therefore, utilizing these invertebrates as a source of collagen presents a valuable opportunity for resource utilization while also helping to mitigate the negative ecological impacts associated with their overabundance. Figure 2 illustrates the diversity of marine organisms that have been recently studied as a source of marine collagen for food application. Table 2 shows the marine collagen sources with their classification, extraction methods, extraction yield, collagen type, and parts that contain collagen.

### 3.1. Collagen from Marine Vertebrates

Fish collagen, primarily obtained from byproducts including fishbone, scales, skins, fins, swim bladder, and cartilage from blue sharks, represents an underutilized resource in the fish processing industry. These byproducts may account for up to 75% of total fish weight. Moreover, recent estimations report that about 25% of the total processed fish is wasted, raising environmental concerns [48]. Furthermore, recent advancements in collagen extraction techniques have demonstrated the feasibility of isolating type I collagen from various fish species, including alu-alu, Asian sea bass, round goby, sole fish, yellowfish tuna, parrotfish, and lizardfish [28,29,32,34,49,50].

Fish skin is recognized for its high content of type I collagen, often reaching a purity level of approximately 70%, which can vary based on species, age, and environmental factors [51]. Typically, the acid-solubilization extraction (ASC) technique is predominantly used for extracting collagen from fish skin due to the marine collagen’s poor water solubility [52,53]. This method has proven effective in extracting acid-soluble collagen from marine species such as chub mackerel (*Scomber japonicas*), Japanese sea bass (*Lateolabrax japonicas*), horse mackerel (*Trachurus japonicas*), and yellowtail snapper (*Ocyurus chrysurus*) [53,54].

Fish scales, another good source of type I collagen, require different extraction conditions compared to fish skin, and it involves higher temperatures (around 16.6–19.03 °C) and longer extraction times (up to 77.5 h), with generally lower extraction yields (~0.13%) compared to fish skin (yields up to 4.3%) [36]. Both salt-solubilization and acid-solubilization extractions have been used to extract collagen from fish scales [10]. Similarly, fish bones, another good source of type I collagen, demand specific collagen extraction conditions. The collagen extraction process from fish bones requires higher extraction temperatures and a shorter time compared to collagen extraction from fish scales and typically results in lower yields compared to collagen extraction from fish skin [10]. Recently, high intensity pulsed electric fields (PEFs) have been used for fish bone collagen extraction with promising results. For example, a study by He et al. [55] combined semi-bionic extraction (SBE) and PEF treatments to isolate collagen from fish bones, achieving an extraction yield of 3.87 mg/mL using a PEF of 22.79 kV/cm. This combined approach was effective in extracting collagen, calcium, and chondroitin from fish bones [55,56].

### 3.2. Collagen from Marine Invertebrates

Marine invertebrates, which constitute a massive portion of marine organisms, offer a vast array of potential collagen sources. Among these, poriferans, commonly known as marine sponges, have a structure like the bones of marine vertebrates. This makes them a noteworthy collagen source [1]. Furthermore, marine collagen has been effectively extracted from various organisms belonging to the Demospongiae class, such as *Chondrosia reniformis* [57]. *Chondrosia reniformis* has been identified as a promising source of type I collagen, with potential applications in foods, cosmetics, and therapeutics [39,57].

The Mollusca phylum, characterized by its diverse range of species, typically features soft-bodied organisms. While most mollusks exhibit a distinct shell, a noteworthy number of species lack this feature. The skin of mollusks, comprising dermis and epidermis layers, contains type I and type II collagen. Recent research has identified the molluscan byssus and cephalopods as potential sources of type I collagen [46,58]. Furthermore, during the processing of crustacean seafood, such as prawns, shrimp, crabs, and lobsters, a substantial volume of waste is generated, primarily composed of carapaces and heads. This waste may account for up to 85% of the total weight of the crustacean and contains from 20 to 40% protein with significant amounts of collagen [59]. However, collagen extraction from crustacean byproducts is challenging due to their high resistance to acid treatment and the presence of non-collagenous proteins. Therefore, enzymatic extraction methods use specific enzymes to break down collagen without compromising its properties. Recently, Hiransuchalert et al. [47] isolated collagen with pepsin from the muscle of several mantis shrimp, such as *M. nepa, H. harpax, E. woodmasoni*, and *O. cultrifer,* and reported that the primary collagen in shrimp muscles was type I collagen.

Echinoderms, exclusive to the aquatic environment, encompass a diverse group of marine invertebrates across five classes, including Crinoidea (sea-lilies), Ophiuroidea (brittlestars), Holothuroidea (sea cucumbers), Echinoidea (sea-urchins), and Asteroidea (starfish). Starfish, known for their high reproductive capacity and as predators in marine ecosystems, are also a good source of marine collagen [60]. Recent studies have demonstrated successful collagen extraction with pepsin from starfish species such as *A. pectinifera* and *A. rubens*, with collagen yields varying from 1.44% to 6.1%, respectively [40,61]. Additionally, type I collagen has also been extracted from sea cucumber (*H. cinerascens*) using pepsin, acetic acid, and a NaCl solution [60]. Moreover, the Coelenterate phylum encompasses a diverse array of marine animals, including hydra, jellyfish, sea pens, and sea anemones. Notably, jellyfish is a good marine collagen source. Like mammalian and other marine collagens, the types of collagens in jellyfish vary depending on the species, origin, and age of the animals. Therefore, collagen from different jellyfish species may show unique functional and/or physical properties [5]. A recent study reported that type I collagen derived from the jellyfish (*Rhopilema esculentum)* closely resembles human collagen, rendering it suitable for a diverse range of biomedical applications [62]. In another study by Chiarelli et al. [44], gelatin powders with exciting gelling properties were developed from salted, dried cannonball jellyfish (*Stomolophus meleagris*) for food applications, demonstrating its potential as a gelling, thickening, or binding agent.

## 4. Production and Processing Technologies of Collagen and Collagen-Based Products

**Figure 3:**
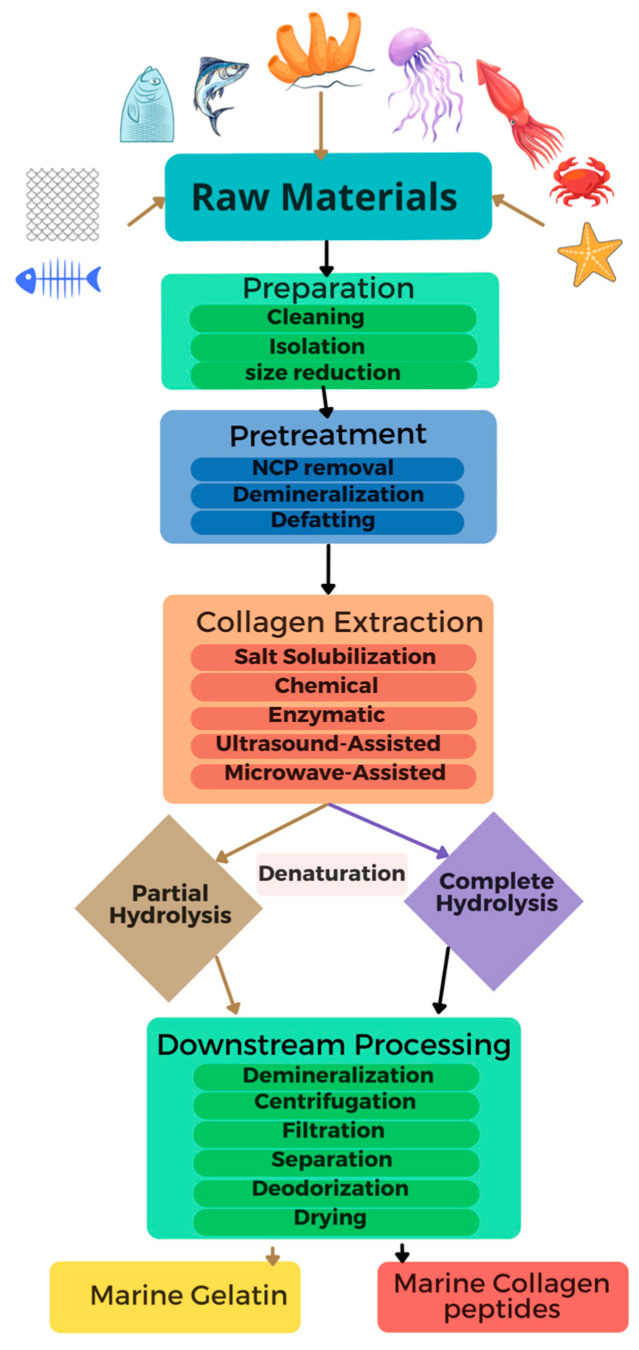
Production and processing diagram of marine collagen and its derivatives.

The production and processing of collagen and collagen-based products encompass several distinct stages, which are essential to ensure the functionality and purity of the final products. These stages typically include preparation and pre-treatment, collagen extraction, denaturation, and downstream processing [42]. The following subsections provide a comprehensive review of the recent advances in the production and processing of marine collagen and its derivatives. Figure 3 illustrates the processing steps of marine collagen-based products (gelatin and collagen peptides).

### 4.1. Preparation of Raw Materials and Pretreatment

The extraction of marine collagen often requires preparation and pretreatment steps to remove impurities and non-collagenous materials, which are crucial for ensuring the purity and quality of the final products [63]. The preparation of the raw materials varies depending on their nature and involves critical processes such as cleaning, the isolation of collagen-containing components, and size reduction. For instance, distinct parts like skins, scales, fins, and bones are separated when processing byproducts due to their diverse collagen compositions and mineral content. Meanwhile, when jellyfish are used as a raw material, a common approach involves separating the oral arms from the umbrellas [64]. The size reduction of raw materials facilitates the removal of contaminants and aids the effectiveness of subsequent pretreatment procedures [65].

Raw materials for collagen extraction often contain contaminants, including non-collagenous protein (NCP), fats, minerals, and pigments, which affect collagen extraction [66]. Thus, one or more pretreatment methods, including non-collagenous protein removal, demineralization, and defatting, are employed to remove these contaminants and increase the extraction yield. These steps are critical to prevent contamination and optimize the extracted collagen’s yield and quality [10]. Generally, either acidic or alkaline pretreatment methods are performed to remove the non-collagenous proteins from the raw materials [19]. Acidic pretreatment with hydrochloric acid (HCl) is more compatible with raw materials containing fewer entangled collagen fibers, such as fish skin [67]. However, alkaline pretreatment, particularly with 0.1 M sodium hydroxide (NaOH) solution, is widely regarded as an effective method for non-collagenous protein removal before collagen extraction because of its substantial swelling properties, which enhances collagen extraction by improving mass transfer in the tissue matrix. Several studies have employed alkaline pretreatment with 0.1 M NaOH solution to remove non-collagenous protein from shortfin Scad waste materials [68], Miiuy croaker scales [69], squid outer skin [58], and hybrid sturgeon skin [70]. Nevertheless, in a study by Liu et al. [71], structural damage was observed in extracted collagen when pretreating the raw materials with a higher concentration (0.2–0.5 M) of NaOH.

Furthermore, raw materials, including fish bone, cartilage, and scales, contain significant amounts of minerals and salt. The demineralization process is used as a pretreatment step to remove these minerals, thereby enhancing the efficiency of the collagen extraction process by creating a porous structure with increased surface area [19,65]. Ethylenediaminetetraacetic acid (EDTA), HCL, and acetic acid are commonly used as demineralization solvents to remove the minerals from the raw materials [11,72]. Among these solvents, 0.5 M EDTA is researchers’ most popular dementalization solvent because of its chelating function [73,74]. Several studies have employed 0.5 M EDTA as a demineralization solvent for collagen extraction from shortfin Scad [68], squid cartilage [75], and Miiuy croaker scales [69].

Defatting is another pretreatment process used to remove fat from high-fat-content raw materials by soaking them in an alcohol solution. Generally, butyl alcohol, ethanol, hexane, and acetone are used to remove fat from raw materials before collagen extraction. For instance, 10% butyl alcohol was used as a defatting solvent in several studies as a pretreatment before extracting collagen from puffer fish skin [76], hybrid sturgeon skin [70], and squid skin [58]. Moreover, Alves et al. [77] used 10% ethanol to remove fat from salmon skin, whereas Zhang et al. [78] used 99.5% ethanol for Bester sturgeon skin.

### 4.2. Collagen Extraction

The poor solubility of collagen in cold water is a well-established phenomenon, primarily due to the existence of strong cross-links within its triple-helix structure. Thus, collagen extraction is performed using a specific solvent, and the extractability of collagen depends on the type and concentration of the solvent used. The most commonly used methods for collagen extraction are salt-solubilization extraction, chemical (acid and alkali) extraction, and enzymatic extraction (pepsin). However, ultrasound-assisted and microwave-assisted extraction are rapidly gaining greater significance for collagen extraction from marine sources. Therefore, the following sections discuss recent developments in marine collagen extraction techniques.

#### 4.2.1. Salt-Solubilization Extraction (SSE)

In collagen extraction, using neutral saline solutions is a common technique, mainly due to collagen’s solubility in salt-containing solutions. Saline solutions, often prepared with sodium chloride, phosphates, citrates, or Tris-HCl, are used to solubilize collagen from fish skin, cartilage, bones, and scales. The salt-solubilization extraction method operates on the principle of salting-out, where the solubility of collagen in a solution decreases with the addition of salt [18]. Adding salt ions to the solution neutralizes the collagen molecules’ surface charge, reducing their electrostatic interactions and causing them to precipitate [47]. Lin et al. [79] used the salting out method with 1.5 M NaCl solution for collagen extraction from bigeye tuna (*Thunnus obesus*) with a yield of 14.14%.

The characteristics of the collagen extracted are significantly influenced by the specifics of the salt-solubilization process. Salt-solubilization extraction is often used with other extraction methods to maximize yield and maintain the desired properties of the extracted collagen. However, the efficiency of salt-solubilization extraction has been questioned, with some studies pointing to its lower efficacy compared to other extraction methods [80]. Therefore, it is rarely used for marine collagen extraction [10]. However, one critical aspect to consider in salt-solubilization extraction is the regulation of salt concentration, as this can affect the solubility and precipitation of collagen. For instance, type I collagen has optimal solubility in salt solutions with concentrations below 1.0 M. In contrast, it tends to precipitate in solutions with concentrations above this threshold [81].

#### 4.2.2. Chemical Extraction

The chemical extraction of collagen is widely popular for producing commercially available collagen-based products. It is often preferred over the salt-solubilization method due to its effectiveness and efficiency [10,67]. This method is primarily divided into two categories: acid and alkali extraction.

Acid extraction is particularly effective for extracting collagen from raw materials having fewer cross-links, as the acid helps to disrupt non-covalent bonds in an organized way [82]. Acid extraction uses organic and inorganic acids to cleave peptide bonds among collagen molecules, thereby facilitating the extraction of collagen strands. In acidic conditions, collagen molecules acquire an increased positive charge, leading to repulsion to tropocollagen molecules (a subunit of polymeric collagen) and thus enhancing solubility [81]. Both organic acids (acetic, citric, lactic, and chloroacetic acid) and inorganic acids (like hydrochloric acid) are utilized for collagen extraction [83]. Organic acids are often more effective in breaking the collagen molecules’ cross-links than inorganic acids, resulting in enhanced collagen extraction [71]. Acetic acid is widely utilized for its ability to modify the electrostatic properties of collagen, thereby enhancing its solubility and extractability [82]. The extraction process generally involves treatment with 0.5 M acetic acid solution with continuously stirring for 24–72 h [80]. Subsequent steps to isolate crude collagen powder typically include, filtration, sodium-chloride precipitation, dialysis, centrifugation, and freeze-drying [67].

The efficiency of acid-based collagen extraction is influenced by the nature of the raw materials. For marine collagen, maintaining a low temperature of approximately 4 °C with continuous stirring for 24–48 h is essential to ensure optimal yield. The concentration and type of acid play a significant role in determining the effectiveness of the extraction process [10,84]. It has been observed that the extraction process involved in low pH and elevated temperatures can produce collagen with a reduced molar mass, which may contribute to the formation of a stronger gel structure [67]. Therefore, the pH of the extraction medium is a critical factor affecting the physicochemical attributes of the extracted collagen. Moreover, the extraction yield often correlates with the duration of the process; however, utilizing multiple successive extraction cycles has proven to be more effective than simply prolonging extraction time [85]. In addition, temperature is another critical factor, with the optimal extraction of acid-soluble collagen typically occurring within the range of 4 to 20 °C, thereby preserving the structural integrity of the collagen [71]. Pal and P.V. [10] showed that collagen extraction with a 6 M HCl solution using a 6 M HCl solution at a temperature between 110 to 120 °C for 18–48 h is a highly effective collagen extraction process.

Alkaline extraction involves the use of basic solutions to eliminate non-collagenous proteins, lipids, pigments, and inorganic substances from materials [10]. The effectiveness of this process largely depends on critical factors such as the duration, temperature, and concentration of the alkaline solution [65]. According to Schmidt et al. [67], employing a 0.05 M NaOH solution is optimal for preserving acid-soluble collagen and maintaining its structural integrity when conducted within a temperature range of 4 to 20 °C. In contrast, the use of higher concentrations of NaOH solutions, particularly at temperatures between 15 and 20 °C, can lead to structural alterations that may diminish the yield of acid-soluble collagen. The alkaline pretreatment is suitable for processing dense and rigid raw materials, as it enhances penetration and facilitates the cleavage of inter- and intra-molecular collagen cross-links [67]. Additionally, strong alkaline agents such as sodium carbonate may be employed in alkaline collagen extraction. These alkali solutions, known for their potent hydrolytic activity, act on collagen fibrils to promote their breakdown and solubilization [10,81]. However, due to the harsh extraction conditions, this collagen extraction method may lead to the degradation of specific amino acids such as serine, cysteine, histidine, and threonine [10].

#### 4.2.3. Enzymatic Extraction

Enzymatic extraction is a highly regarded biological method for commercial collagen production due to its specificity and minimal impact on collagen structure [67]. This method maximizes collagen yield through higher reaction selectivity, differentiating it from chemical extraction. Furthermore, enzymatic extraction presents several benefits over conventional chemical extraction, including enhanced specificity, a more controlled degree of hydrolysis, milder reaction conditions, lower residual salt content in the final products, reduced waste generation, and improved collagen yield [86]. Although enzymatic extraction is generally more costly, its numerous benefits make it a valuable approach to collagen production. Moreover, combining enzymatic with chemical extraction methods has been shown to maximize collagen yield and improve the overall quality of the extracted collagen [87]. The feasibility of acid–enzyme, alkali–enzyme, and acid–alkali–enzyme extraction for the commercial production of marine collagen has been reported. For instance, 0.5 M acetic acid with different concentrations of pepsin (0.05–10.0%) has already been reported for collagen extraction from other marine species [33,68,88].

Several proteolytic enzymes have been utilized for the enzymatic collagen extraction, including those derived from plants (bromelain, papain, and ficin), animals (trypsin and pepsin), and other sources (collagenase, proteinase K, alcalase, nutrase, flavourzyme, and protamex). Pepsin is a widespread enzyme due to its ability to hydrolyze the non-helical peptide chain without affecting the helical structure [71,81]. Papain is another enzyme noted for its effectiveness in cleaving collagen. Jin et al. [89] successfully combined microwave radiation with papain to obtain collagen from sea cucumber (*Acaudina molpadioides*).

The use of exogenous enzymes such as papain and trypsin has been recently evaluated because they can regulate hydrolysis more efficiently and shorten the collagen extraction time compared to other methods [89]. Conversely, endogenous enzymes such as cysteine proteinase, serine proteinases, and matrix metalloproteinases, isolated from different sea cucumber species, are effective in isolating marine collagen [90,91,92]. Yan et al. [93] found that serine proteinases derived from sea cucumber can break down collagen cross-links; these findings are supported by another study using trypsin-assisted collagen extraction from *Stichopus japonicus* [90].

#### 4.2.4. Ultrasound-Assisted Extraction (UAE)

UAE has emerged as a promising alternative to traditional collagen extraction methods, offering advantages such as reduced processing times and increased yields [94]. Ultrasonic treatments (frequencies higher than 20 kHz with an amplitude of 20–80%) have been effective at extracting collagen with high yields due to the facilitation of mass transfer in a wet medium [67,95]. The ultrasonication process involves the application of ultrasonic vibrations to agitate particles within a material. A key phenomenon in UAE is cavitation, which occurs when ultrasound waves create microscopic bubbles in a liquid. These bubbles grow and eventually collapse, generating localized high-pressure and temperature conditions [96,97]. Cavitation can disrupt biological tissues and cellular structures, enhancing collagen extraction [95]. Moreover, the amplitude and application time of ultrasound treatment greatly influence the extracted collagen yield [98]. In a recent study, Pezeshk et al. [99] studied the impact of ultrasound (300 W, 0–25 min) treatment on the extractability of native collagen from byproducts, compared with the conventional chemical (acid) extraction method. The study revealed that ultrasound treatment significantly increased collagen yield, producing 2.7 times more collagen than the conventional acid extraction method. Additionally, the study reported improvements in hydroxyproline content and thermal stability, with the native triple-stranded helical structure of collagen remaining intact due to the controlled ultrasound application. Furthermore, Song et al. [100] demonstrated that UAE yielded significantly higher collagen extraction than chemical extraction methods, emphasizing its potential for commercial collagen production from fish skin.

Ultrasound-assisted extraction is often employed with chemical or enzymatic extraction methods to increase the collagen extraction yield and reduce the extraction time [100]. Ali et al. [98] combined the ultrasound treatment (20 kHz) with the conventional chemical (acid) and enzymatic extraction process and found that combining ultrasound treatment with conventional extraction methods increased the yield of extracted collagen compared to the conventional methods. The author further reported that the increased amplitude of ultrasound treatment (20–80%) further increased the collagen yield in both acid-soluble and enzyme-soluble collagen extractions. In a similar study, Lee et al. [101] evaluated the effects of enzymatic, ultrasound, and combined enzymatic–ultrasound extraction on the production of collagen hydrolysate from Alaska Pollock (*Theragra chalcogramma*) skin. The study reported that the combined enzymatic–ultrasound approach was the most efficient method for producing collagen hydrolysate, surpassing the yields of either treatment alone.

Despite these advantages, the ultrasound-assisted collagen extraction method poses some challenges, including the disruption of hydrogen bonds between the collagen chain and temperature increase during ultrasonication [102]. Therefore, further research is necessary to fully understand the effects of UAE on the structural integrity of collagen and to optimize the process for commercial extraction.

#### 4.2.5. Microwave-Assisted Extraction

The microwave-assisted extraction process relies on using electromagnetic waves to disrupt cellular structures, thereby enhancing the extraction process [103]. This method enables microwave radiation to penetrate deep into protein matrices, loosening the cellular structures and facilitating the release of target molecules from the cell matrix [89]. In the context of collagen extraction, this microwave-assisted process is typically complemented by subsequent enzyme hydrolysis, using either acidic or enzymatic methods to complete the breakdown of collagen [104]. The effectiveness of this combined approach has been demonstrated in studies such as those by Jin et al. [89], who explored the enzymatic hydrolysis of collagen from sea cucumber, showing that microwave energy significantly boosts the hydrolysis process, leading to an efficient decomposition of collagen fibrils.

A summary of the pros and cons of marine collagen extraction methods based on efficiency, cost, energy requirements, and impact on collagen structure is presented in Table 3.

### 4.3. Denaturation of Marine Collagen

**Figure 4:**
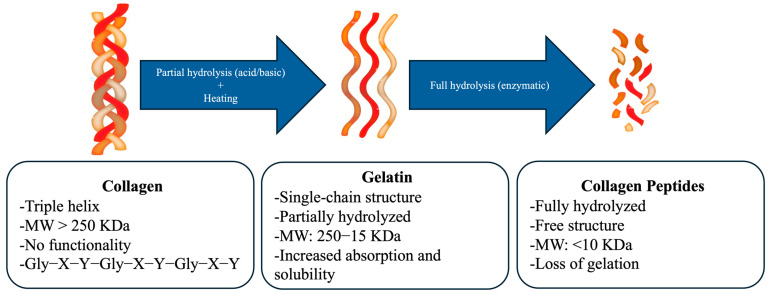
Denaturation of marine collagen.

The solubility of marine collagen in chilly water is typically low, primarily due to the presence of strong cross-links and hydrogen bonds within its triple-helix structure. This feature limits its applications that require high water solubility [5]. Marine collagen is often denatured using chemical (acid and alkaline hydrolysis) and biological methods (enzymatic hydrolysis) to increase water solubility. Each denaturation technique is tailored to the specific characteristics of collagen from various sources. Notably, the denaturation process enables the production of various collagen formulations, such as biodegradable films, tablets, granules, and sponges [10]. When the triple-helix structure of marine collagen is divided into single-strand polypeptides via heat denaturation or partial chemical or enzymatic hydrolysis, water-soluble gelatin is formed, which can be used as a functional ingredient in foods. On the other hand, the complete hydrolysis of collagen produces collagen peptides with higher absorption capability and bioavailability. Figure 4 shows an overview of the denaturation process of marine collagen.

According to Pal and P. V. [10], hydrolysis breaks the hydrogen bonds that stabilize the collagen triple helix and form gelatin (a water-soluble hydrocolloid of high molecular weight that does not exist in nature). Generally, acid hydrolysis produces type A gelatin, while alkali hydrolysis produces type B gelatin [105]. Gelatin is a mixture of single multistranded polypeptides with a left-handed helix conformation containing 50–1000 amino acids. Moreover, gelatin closely resembles the chemical composition of collagen, with molecular weight ranging from 3 to 200 KDa depending on the raw materials and extraction process [1]. Like collagen, gelatin contains Gly (every third residue), Pro, and Hyp residues. Marine gelatin’s typical amino acid composition is -Ala-Gly-Pro-Arg-Gly-Glu-Hyp-Gly-Pro-, which may vary depending on the collagen source and processing techniques [1]. Interestingly, partially hydrolyzed collagens or gelatins show gelling, foaming, emulsifying, stabilizing, and film-forming properties, which make them suitable for several food applications [63,106]. For example, Duan et al. [107] reported adding channel catfish skin gelatin into ice cream, resulting in a thicker and smoother texture than ice cream made with bovine gelatin. Also, a low concentration (<0.04%) of channel catfish skin gelatin effectively reduced the cloudiness and clarified beer and wine. In addition, Chiarelli et al. [44] developed novel gelatin powders from salted, dried cannonball jellyfish with promising gelling and thickening properties.

Complete hydrolysis (via enzymatic or chemical means) produces collagen peptides. Collagen from fish (Alaska pollock, shark, and tilapia), byproducts, and jellyfish have produced marine collagen peptides [108]. Individual enzymes such as alcalase, papain, pepsin, and collagenase are the most effective enzymes for producing collagen peptides [108,109]. However, using a mixture of proteases has proven more effective [1]. After hydrolysis, ultrafiltration and chromatography are subsequently used to fractionate the resultant peptides. Collagen peptides show higher solubility, digestibility, and bioavailability than native collagen and gelatin. However, collagen peptides do not have thickening or gelling properties [63].

### 4.4. Downstream Processing

Marine collagen and its derivatives can undergo numerous downstream processes to produce high-quality, shelf-stable products. The conventional downstream processing methods for manufacturing collagen-based products include demineralization, sonication, centrifugation, filtration, separation by chromatography, deodorization, and drying [5]. In general, collagen and hydrolyzed collagen may contain various amounts of minerals, such as NaCl; hence, employing a demineralization step as a downstream process is essential to increase the quality of the final products. Demineralization has been shown to positively affect the physical and functional characteristics of hydrolyzed jellyfish collagen, influencing factors such as color, bloom strength, apparent viscosity, yield stress, and crude protein without altering the amino acid profile or the isoelectric point of the final products [110]. Furthermore, sonication may accelerate desalination and improve the final product’s functional and physicochemical properties [88,111]. After collagen denaturation via hydrolysis, centrifugation and filtration are commonly used to isolate specific collagen peptides based on their solubility and size [112]. Separation via chromatographic methods (such as gel filtration, reserved-phase, ion exchange, and size exclusion chromatography) is an additional process that may be used to purify collagen-based products. Moreover, certain marine collagens, such as jellyfish collagen, contain small amounts of polyunsaturated fatty acids, which are prone to produce rancid odors when oxidized [113]. Deodorization is often performed to remove these volatile and off-flavors from jellyfish collagen. Wang et al. [114] recently reported that physical (absorbance, ultrafiltration, masking, steaming, irradiation, and embedding), chemical (acid/alkaline, ozone, antioxidants, and Maillard reactions), and biotechnological (yeast and lactic acid fermentation) methods are promising methods to remove off-flavors (mainly alkanes, alcohols, aldehydes, ketones, acids, amines, sulfur compounds, furans, pyrazines, thiazoles, and indoles) from fish collagen peptides.

Another crucial downstream process in creating shelf-stable collagen-based products is drying. The drying process impacts the functionality of final products. Generally, gelatin’s functional properties depend on its three-dimensional structure [115]. During the drying process, heat may induce cleavage in the covalent and non-covalent bonds and change the physicochemical and structural properties of gelatin, which subsequently influence the functional properties of gelatin, such as gelation, solubility, emulsification, and fat and water absorption capability [115]. Notably, the magnitude of these changes depends on the drying conditions and methods used. The researchers revealed that spray-drying is one of the most efficient processes to enhance protein solubility, emulsifying, and foaming characteristics of marine gelatin powders compared to freeze and vacuum drying. Nonetheless, the gel strength of the gelatins remained largely unaffected by the drying method used [116]. Mad-Ali et al. [117] reported that freeze-drying and spray-drying have similar effects on the gelation properties of gelatin. Additionally, Rasli and Sarbon [118] reported that freeze-drying improved the foaming property in gelatin, while vacuum drying enhanced the stability and fat-binding ability. Moreover, a study recently reported that combining infrared radiation and convection hot air drying is effective for drying fish gelatin [119].

Cross-linking is a crucial downstream process that significantly enhances marine collagen’s thermal stability and mechanical properties by promoting chemical and physical interactions between collagen fibrils. Type I collagen, known for its relatively poor mechanical properties and strength, can benefit significantly from cross-linking. This process improves collagen’s thermal and mechanical performance and makes it suitable for applications such as cartilage tissue engineering (CTE), particularly in the form of scaffolds [120]. Cross-linking can be achieved through physical, chemical, and biological processes. Physical cross-linking is commonly performed using dehydro-thermal treatment (DHT) or ultraviolet irradiation (UVI). DHT involves the removal of water from the collagen at high temperatures under vacuum conditions, while UVI utilizes ultraviolet radiation to induce cross-linking [54,121]. In contrast, chemical cross-linking interconnects collagen fibrils to extend the ultrastructure duration and improve mechanical strength by reducing enzymatic degradation in vivo [122]. Glutaraldehyde, N-hydroxysuccinimide, and 1-ethyl-3-(3-dimethalamino-propyl are commonly employed as chemical cross-linkers in collagen; these compounds are cytotoxic and require thorough cleansing with deionized water before use to mitigate their harmful effects [123]. Due to the cytotoxicity of chemical cross-linkers and the limitation of cross-linkers, biological cross-linkers have developed in recent years as safer alternatives. Biological cross-linkers, such as Genipin, transglutaminases, tyrosinase, lysyl oxidase, phosphatases, horseradish peroxidase, and hydrogen peroxide, have demonstrated effectiveness in cross-linking type I collagen. Genipin, for instance, reacts with the amino group of amino acids to produce dark blue pigments, which improve mechanical strength and increase resistance to the enzymatic degradation of type I collagen-based scaffolds [124]. The cross-linking mechanism of Genipin involves forming an aldehyde group with a C3 carbon atom and substituting an ester group with a secondary amide bond, further contributing to its efficiency as a cross-linker. Recently, a study by Chiarelli et al. [125] cross-linked hydrolyzed jellyfish gelatin powder with pomegranate peel polyphenol powder, along with adjusting pH, solids concentration, and maturation temperatures, which increased the bloom strength of the hydrolyzed jellyfish gelatin and increased the resistance against the deformation of the gelatin gels.

Downstream processes significantly influence the physicochemical properties and determine the final quality and functionality of extracted collagen. For instance, freeze-drying preserves the triple-helix integrity, and thereby helps to maintain gelation properties and enzymatic digestion, while spray-drying may lead to partial denaturation due to heat exposure [116]. Similarly, chemical or physical cross-linking are often applied to enhance mechanical strength and thermal stability, which may reduce solubility and enzyme degradability, thereby affecting collagen’s application in a biomedical or nutraceutical context [122]. Moreover, enzymatic hydrolysis is used to produce collagen peptides with improved absorption, which reduces gelling ability due to the disruption of the triple-helical structure. Therefore, a careful balance must be maintained between structural modification and functional retention depending on the intended end use.

### 4.5. Emerging Analytical Techniques for Molecular Characterization of Collagen

Recently, advancements in analytical techniques have helped to comprehensively characterize the molecular structure of collagen. For example, Fourier-transform infrared spectroscopy (FTIR) remains a foundational tool for assessing the secondary structure of collagen, particularly the presence of α-helix, β-sheet, and random coil arrangements through the analysis of amide I and II bands [65]. Complementarily, circular dichroism (CD) spectroscopy provides sensitive measurements of collagen’s triple-helical content and thermal stability by detecting conformational changes in the far-UV region [126]. Furthermore, nuclear magnetic resonance (NMR) spectroscopy, especially solid-state ^13^C and ^15^N NMR, has emerged as a powerful method to investigate atomic-level interactions and the dynamics of collagen matrices, particularly in biomaterials research [127]. Additionally, small-angle X-ray scattering (SAXS) and wide-angle X-ray diffraction (WAXD) techniques allow the elucidation of hierarchical structures, including fibril alignment and crystallinity, essential for correlating collagen structure with mechanical performance [128]. More recently, cryogenic transmission electron microscopy (cryo-TEM) and atomic force microscopy (AFM) have enabled high-resolution visualization of collagen fibrils and supramolecular assemblies, providing critical insights into the effects of processing or cross-linking on collagen morphology [129]. These analytical tools provide a robust framework for characterizing the structure–function relationship of collagen and facilitating the design of next-generation collagen-based materials for food and biomedical applications.

## 5. Nutritional Composition and Health Benefits of Marine Collagen

Marine collagen comprises three polypeptide chains, each containing thousands of amino acids, including glycine, proline, and hydroxyproline [1]. Glycine is one of the most prevalent amino acids in marine collagen, and it may constitute up to 30% of marine collagen’s amino acid content [130]. While the amino acid composition of marine collagen shares similarities to mammalian collagen, it generally has lower levels of imino acids, such as proline and hydroxyproline, than mammalian collagen [22,131]. However, marine collagen derived from cold-water fish often shows higher levels of serine and threonine content than mammalian collagen [132]. Overall, marine collagen contains some essential amino acids that are important for human health [10]. Damodaran and Parkin [133] reported that type I collagen typically consists of glycine (33%), proline (12%), alanine (11%), hydroxyproline (10%), and small quantities of glutamic acid and aspartic acid. According to Ahmed et al. [134], the native marine collagen molecule’s main function is structural and does not show any type of bioactivity. However, when they are isolated by different mechanisms, they show biological functions, including antioxidant, antimicrobial, anti-inflammatory, antifreeze, angiotensin-I converting enzyme (ACE-I) inhibition, anti-aging, wound healing, and anticoagulant activities [135,136,137,138,139,140].

As we age, our body’s collagen production decreases, impacting bones, joints, and skin. Dietary marine collagen-based products may offer several health benefits, including enhancing skin health, strengthening bones and connective tissues, and promoting gastrointestinal wellness [141]. For instance, Liu et al. [104] suggested that collagen peptides minimize skin aging by promoting collagen synthesis, preventing collagen breakdown, and reducing oxidative stress in skin cells. Moreover, Ito et al. [142] reported that dietary supplementation of fish collagen peptides and ornithine (CPO) reduced trans-epidermal water loss and pore count while increasing skin elasticity in healthy participants. De Luca et al. [143] reported that consuming 570 mg of collagen peptides derived from the skin of deep-sea fish combined with plant-derived antioxidants resulted in increased skin thickness in study participants. Similarly, Evans et al. [144] evaluated the safety and effect of dietary marine collagen from freshwater fish skin on the skin of women aged 45–60 years. The study reported that after three months of supplementation, participants’ wrinkles were reduced by 35%. Furthermore, it was noted that the supplements were safe and well-tolerated. In addition, it was observed that there were improvements in hydration (14%), radiance (22%), and skin firmness (25%) when compared to the placebo group. Nonetheless, dietary marine collagen supplementation also promotes bone health. For example, marine collagen peptides have been found to aid in calcium and zinc absorption, crucial for bone health and osteoporosis prevention [145].

On the other hand, marine collagens showed promising results in cartilage regeneration, which is crucial for managing osteoarthritis (the loss of cartilage) [146]. Bourbon et al. [147] examined the impact of three collagen peptides derived from fish skin and cartilage on the degradation of chondrocytes. The study revealed that collagen peptides at concentrations of 0.5, 50, and 100 μg/mL significantly increased type I and II collagen levels. Moreover, Ornishi et al. [148] reported protective effects against osteoarthritis in rabbits treated with fish collagen peptide and glucosamine.

Most of the reported health benefits of marine collagen are primarily based on preclinical models, with only a few small-scale human studies available. Additionally, the existing human trials often have significant limitations, including small sample sizes, short treatment durations, and a lack of diverse populations. These issues make it challenging to draw definitive conclusions about the effectiveness and safety of marine collagen for specific health outcomes, such as skin elasticity and joint health.

## 6. Food Applications of Marine Collagen-Based Products

Marine collagen and its derivates are used as food additives, functional ingredients, and film-forming agents in food and beverage industries. Notably, the functionality of marine gelatin and collagen peptides depends on their rheological and thermal properties [5]. Gelation is a crucial functional property of collagen that significantly influences the texture, mouthfeel, and structural integrity of various food products. Marine gelatin can undergo gelation when dissolved in water, forming a thermo-reversible three-dimensional network that contributes to a soft and elastic texture [149]. The gelation behavior is closely related to the molecular structures of the collagen peptide chains and their amino acid composition, with proline and hydroxyproline content playing critical roles in stabilizing the triple-helix structure, affecting gel strength and melting temperature [150]. In addition to gelation, the emulsifying properties of collagen peptides and gelatin are essential for stabilizing oil-in-water emulsions in food systems. The amphiphilic molecular structure of marine collagen effectively forms stable emulsions with desirable homogeneity, texture, and shelf life. These characteristics have potential applications in emulsified food products such as sauces, salad dressings, and processed meats [151]. Furthermore, the presence of specific hydrophobic amino acids enhances interfacial adsorption, contributing to emulsion stability. These functional properties improve the sensory and physicochemical attributes of food products. Table 4 illustrates the food applications of marine collagen.

### 6.1. Collagen As a Viscosity, Texturing, and Stabilizing Agent

Gelatin can form thermo-reversible hydrogels with melt-in-the-mouth properties [157]. Moreover, it can be used in confectionaries, reduced-fat condiments, baked goods, and meat. Marine gelatins can enhance the texture of meat products, can function as an emulsifier in acidic foods, and can be incorporated into beverages for nutritional and functional benefits [157]. Hydrogels produced with marine gelatin often show lower gelling and melting temperatures due to their lower proline and hydroxyproline content compared to mammalian gelatins. Furthermore, gelatin gels from cold-water fish have lower gelling and melting temperatures than gelatin gels obtained from warm-water fish [158]. Recent efforts to improve the gelling properties of marine gelatins include adding natural ionic polysaccharides such as gum arabic, pectin, sodium alginate, and k-carrageenan [159,160]. For instance, Sinthusamran et al. [161] improved the gel strength and melting temperature of fish gelatin gels by adding 50% k-carrageenan. Anvari and Joynar [159] added gum Arabic into fish gelatin at a lower pH (5.0) and increased the gel strength and structural stability of the resultant gels. Moreover, different types of marine gelatins (e.g., cold- and warm-water fish gelatins) have been combined with, or added to, proteins, salts, and plant phenolics to improve gelling properties [162]. Meanwhile, Yan et al. [163] used gallic acids and rutin as cross-linking agents and improved the elastic modulus and viscous modulus of Walleye pollock gelatin gel.

### 6.2. Collagen As a Functional Ingredient in Beverages

The incorporation of collagen in beverages has sparked the attention of the food industry. Soy, cocoa, and coffee beverages as well as fruit juices have been fortified with collagen [162]. Collagen can also be used to clarify cloudy alcoholic beverages by promoting the aggregation and sedimentation of yeast and other insoluble particles. In addition, Bilek and Bayram [153] demonstrated the effective incorporation of hydrolyzed fish collagen into orange, apple, and white grape juice to improve their dietary and functional characteristics. Moreover, hydrolyzed collagen improves the nutritional and functional characteristics of orange juice and the physicochemical and microbiological properties of fermented dairy products [164]. Furthermore, due to the low molecular weight, fish collagen peptides have been incorporated into beverages due to their high solubility and low viscosity, precipitation, and turbidity when added in lower amounts (2–3%). However, adding more than 30% of hydrolyzed fish collagen in beverages can significantly increase viscosity, affecting quality and functionality [153].

### 6.3. Collagen As a Film-Forming Agent

Collagen-based edible films are used extensively in the food industry as a protective barrier against moisture and oxygen, hence enhancing the longevity of food products. In addition, collagen-based edible films and coatings can be applied to meat and fish products to improve juiciness and minimize cooking shrinkage. For instance, Liu et al. [165] reported that gelatin from blue shark skin showed film-forming properties, which can be used for the preservation of red meat. Moreover, marine collagen is used as a promising biomaterial for food packaging and as a fat substitute in processed meats [166]. Furthermore, the film-forming capabilities of fish gelatin are effective in the encapsulation of drugs and other bioactives, such as antioxidants and antimicrobials [18].

On the other hand, biodegradable films made with collagen are emerging in the food industry due to their low environmental impact. Several studies evaluated the production of marine gelatin-based biodegradable packaging from jellyfish [167,168], puffer fish [169], and starry triggerfish skin [170]. However, marine gelatin-based films often show lower mechanical and barrier properties compared to those made with mammalian collagen due to their highly hygroscopic nature [171]. Notably, the film-forming ability of marine collagen and gelatin mainly depends on the molecular weight distribution and amino acid composition [18]. Therefore, current trends in designing marine gelatin-based biodegradable food packaging are focused on developing biodegradable films with enhanced mechanical and barrier properties by combining marine gelatins with other biopolymers, lipids, polysaccharides (chitosan, gellan, pectin, and konjac glucomannan), protein isolates, hydrophobic and hydrophilic plasticizers, alcohol, and cross-linking agents (glutaraldehyde and TGas) [171].

### 6.4. Collagen Peptides (CPs)

Collagen peptides can be used as food additives and functional ingredients. As food additives, collagen peptides can function as antioxidants in lipid foods, emulsifiers in emulsion-based foods, fat substitutes in low-fat meat products, and clarifiers in beverages. The addition of fish collagen peptides has reduced lipid oxidation in meatballs [172]. Fish collagen peptides can extend the shelf life of emulsion-based foods, such as butter and chocolate [152]. Moreover, they can be added to beverages to improve nutritional and functional properties [153]. Furthermore, the addition of marine collagen peptides in acai pulp and cheese showed positive viscosity changes and higher sensorial acceptability, even after 28 days of storage [173]. Additionally, marine collagen peptides have been added to soups to enhance viscosity, functional properties, and antioxidant activities [155].

Marine collagen peptides can also be used as functional ingredients to improve skin health. For example, fish skin collagen peptides promote wound healing by adjusting inflammatory reactions and increasing wound angiogenesis and collagen accumulation [174]. In addition, collagen peptides from silver carp skin have been used to repair photoaging skin [175]. A recent study has reported that collagen peptides promote type I collagen synthesis, which helps to improve skin health [176].

### 6.5. Other Applications

Collagen peptides can be used as effective food cryoprotectants. For instance, marine collagen peptides from shark skin could be used as cryoprotectants in surimi to prevent protein denaturation of surimi during the freezing and thawing process [177]. Moreover, collagen peptides from carp skin could inhibit the ice crystal growth in ice cream during frozen storage and reduce melting, providing a smooth and creamy texture [133]. Furthermore, collagen peptides from jellyfish have shown antiviral, antimicrobial, antineoplastic, and fungistatic properties in several studies [110,138,139].

### 6.6. Case Study for Potential Marine Collagen Valorization: From Sourcing to Market Application

Marine collagen is rapidly emerging as a sustainable and functional ingredient in nutraceuticals, cosmetics, and biomedical applications due to its superior biocompatibility, biodegradability, and low immunogenicity [5]. A concrete example of successful marine collagen utilization may involve the valorization of cannonball jellyfish (*Stomolophus meleagris*) caught off the southeastern coast of the United States. The process may begin with sustainable sourcing, where jellyfish are harvested seasonally from underutilized fisheries, helping mitigate overfishing pressure on traditional marine stocks. Then, fresh jellyfish may undergo collagen extraction and hydrolysis, typically involving an initial acid extraction using organic acid or proteolytic enzymes to yield acid-soluble collagen (ASC) or enzyme-soluble collagen (ESC) [178,179,180,181].

Following extraction, purification may be achieved through dialysis and centrifugation, which facilitate the separation of residual salts and peptides. Then, drying via spray- or freeze-drying can be used to obtain collagen in powder form, maintaining its bioactive conformation and extending shelf life [182]. In formulations, marine collagen can be incorporated into functional foods or dietary supplements. For example, powdered jellyfish collagen peptides (JCPs) can be incorporated into sachets or beverages, targeting skin health and joint support applications. Their high solubility and bioavailability, combined with a favorable amino acid profile—particularly glycine, proline, and hydroxyproline—make them attractive alternatives to bovine or porcine collagen [5].

Market application is exemplified by brands such as Vital Proteins and Skinade, which have introduced marine collagen-based supplements aimed at the health-conscious consumer base. These products claim to support dermal regeneration and aging-related collagen loss, supported by clinical studies linking collagen intake to improved skin elasticity and hydration [183]. Furthermore, regulatory approval for marine collagen in dietary supplements in the U.S. and EU markets has lowered entry barriers, facilitating commercialization. Notably, marine collagen derived from jellyfish provides a unique advantage: lower thermal stability (~24–30 °C), enabling rapid enzymatic hydrolysis into bioactive peptides during digestion, thereby enhancing bioactivity [5]. Additionally, the use of marine collagen aligns with global sustainability goals by valorizing seafood byproducts and reducing reliance on terrestrial animal sources.

This proposed approach provides a full value chain from sustainable jellyfish harvesting to high-purity collagen powder production and demonstrates how marine resources can be effectively leveraged for health and wellness markets. The integration of advanced bioprocessing technologies, coupled with strategic formulation and market positioning, offers a replicable model for scaling up marine collagen applications.

## 7. Safety and Quality Control

The quality attributes of collagen and its derivatives are of the utmost importance in ensuring the anticipated benefits and their safety. Marine collagen-based products have been utilized for extended periods with minimal side effects reported. However, several commercially available collagen-based products may have different safety concerns. Some studies have recently documented the incidence of allergic reactions to certain collagen-based products, specifically those derived from fish collagen. However, the results of these investigations are inconclusive, and perspectives about the significance of fish collagen as an allergen are still up for debate [184]. Due to the limited understanding of its allergenic potential, fish collagen has not been incorporated into diagnostic examinations for fish allergy. Furthermore, fish collagen is not registered yet as an allergen by the World Health Organization and International Union of Immunological Societies (WHO/IUIS) Allergy Nomenclature Sub-Committee (www.allergy.org, accessed on 20 March 2025). In addition, several studies have documented that marine collagen derived from invertebrates, such as jellyfish, exhibits minimal allergenicity [5,185]. Furthermore, marine collagen and collagen-based products have minimal contaminants and toxins, as most of them are eliminated during the production of collagen-based products. Downstream processing used for marine collagen production can significantly reduce the allergenic potential of collagen-based ingredients. For instance, enzymatic hydrolysis, which breaks collagen down into low-molecular-weight peptides, has been shown to decrease allergenicity by disrupting conformational epitopes that are recognized by immunoglobulin E (IgE) antibodies [186]. Additionally, ultrafiltration and purification steps can remove residual non-collagenous proteins and lipid contaminants that may act as allergens. These techniques not only enhance the safety profile of marine collagen but also improve its suitability for use in hypoallergenic formulations.

The quality of collagen and its derivatives depends on their peptide profile, which demonstrates the consistency of the manufacturing process [187]. Therefore, it is imperative to rely on the most suitable analytical methodologies to evaluate the quality attributes of the peptides. Size exclusion chromatography (SEC) is a frequently utilized technique for assessing the polydispersity of collagen peptides [188]. The comprehensive SEC profiles are capable of detecting minimal variations and determining the permissible range of discrepancies among batches, hence establishing the consistency of the manufacturing process [189]. However, advanced tools, such as mass spectrometry (MS) and nuclear magnetic resonance (NMR), are more precise, accurate, and efficient for determining the composition and identity of the components in complex mixtures. Differential scanning calorimetry (DSC) is another analytical technique used to determine the heat stability of collagen [190]. Furthermore, Raman spectroscopy can be used to monitor the pyridinoline trivalent collagen cross-links extracted from mineralized tissues [191]. The implementation of these quality control methods can maintain the uniformity in physical properties, bioactivity, and safety profile of collagen and its derivatives. The molecular weight distribution, hydroxyproline content, amino acid profile, and peptide fingerprinting can be monitored using high-performance liquid chromatography (HPLC), FTIR, and/or SDS-PAGE, allowing manufacturers to detect batch-to-batch variability and adjust processing conditions accordingly. Furthermore, standardizing raw material sources, extraction protocols, and downstream processing will enhance the quality consistency of the collagen and its derivatives.

## 8. Consumer Acceptance and Market Trends

Health-conscious consumers are including collagen-based supplements in their diets due to their health benefits and functional properties [192]. Marine collagen-based products are increasingly preferred by modern consumers compared to mammalian collagens, mostly due to religious, environmental, and health considerations. Muslims and Jews prefer to use marine collagens since some of them can be certified as kosher and halal. In addition, consumers are considering marine collagen as a safer alternative to its mammalian counterpart due to the transmission of BSE and FMD [2]. Despite the increasing popularity of marine collagens, studies examining consumer perceptions of those products are scarce. According to Bhagwat and Dandge [193], adding marine collagen to paneer cheese is acceptable to customers. However, a separate study discovered that the presence of a fish-like aroma and flavor had a negative effect on the overall desirability of marine collagen, rendering it unacceptable when consumed with water. The researchers also proposed masking the unpleasant taste of marine collagen when including it in smoothies or other food items. Flavor-masking techniques, such as microencapsulation, enzymatic treatment, or the incorporation of natural flavor enhancers and odor absorbers, can be developed to mitigate the fishy smell and off-flavors of marine collagen to enhance consumer acceptability [194]. Moreover, refining extraction and purification processes for reducing residual lipids and volatile compounds can significantly improve the sensory profile and consumer acceptance of marine collagen. Additionally, consumer awareness and education about the health benefits, sustainability, and ethical advantages of marine collagen can help to increase the consumer acceptance of marine collagen.

## 9. Challenges and Future Direction

Marine collagen has been gaining popularity in foods and nutritional supplements because of its potential health benefits, religious advantages, and sustainability. However, several challenges and limitations remain in utilizing marine collagen in terms of production, sensory, and textural characteristics. Marine collagens are regarded as having inferior rheological capabilities and thermal stability compared to their mammalian counterparts. These characteristics are primarily determined by their amino acid contents [18]. Furthermore, the aroma and taste of marine collagens often limit consumer acceptability due to their fishy, sour, bitter, and salty characteristics [192]. For instance, using fish gelatin in mildly flavored food products might cause an unpleasant fishy odor, which negatively affects consumer acceptability. In addition, the soaring prices of marine collagen-based products pose a substantial barrier to increasing consumer demand. The commercial production of marine collagens is time-consuming, laborious, and inefficient. Therefore, the industrial manufacturing of marine collagen is challenging [8]. Another challenge in the manufacturing process of marine collagen is the inadequate accessibility of suitable raw materials with consistent quality. Typically, the majority of marine collagen is derived from underused fish and fish byproducts. Producers often face challenges in extracting high-quality collagen due to insufficient amounts of raw materials on a regular basis.

Further research and innovation in marine collagen should focus on addressing existing obstacles linked to marine collagen manufacturing and expanding its utilization. Researchers might explore alternative, sustainable, and innovative sources of marine collagen that go beyond the conventional fish species. Seafood byproducts, such as fish skin, bones, and jellyfish, offer a sustainable and low-cost source of marine collagen. Utilizing these materials reduces waste, adds economic value to underutilized resources, and provides an eco-friendly alternative to mammalian collagen, aligning with circular bioeconomy principles and supporting environmentally conscious biomaterial production.

Exploring marine collagen from a range of marine animals, such as vertebrates and invertebrates, may prove to be a crucial approach to discovering novel sources of marine collagen. In addition, researchers should investigate various methods of flavor masking, such as microencapsulation, nanoencapsulation, and polymer coating, to address the unpleasant fishy taste or odor commonly associated with marine collagen. This would enhance its acceptability and increase its food applications. In addition, new studies should focus on creating novel formulations and delivery systems for marine collagen to improve its bioavailability, stability, and incorporation into functional food and nutraceutical products. Moreover, interdisciplinary research, such as biotechnology, material science, food science, and marine biology, should be integrated to effectively address the existing challenges related to extraction efficiency, product standardization, environmental sustainability, and market scalability.

## 10. Conclusions

Marine collagen offers a promising alternative to traditional collagen sources for food and supplement applications due to its sustainability, functionality, and compatibility with various dietary preferences and restrictions. The abundance of marine organisms (vertebrates and invertebrates) and innovative production techniques have fueled the rapid growth of marine collagen research. Consequently, studies on new marine collagen sources, extraction, processing, and applications led to significant advances. This review has focused on the current state of knowledge on marine collagen and its derivatives, including marine sources, extraction methods, digestion, downstream processing techniques, and its application in foods and supplements. The ability to form a thermo-reversible gel with melt-in-the-mouth properties, as well as gelling, emulsifying, stabilizing, and film-forming ability, triggered the extensive use of marine collagen and its derivatives in the food and supplement industries. However, challenges, including unpleasant sensory characteristics (odor, flavor, and color) and inferior rheological, thermal, and film-forming properties, hinder marine collagen’s widespread use in food and supplement industries. Future studies should focus on optimizing extraction processes to improve extraction yield and quality, enhancing sensory characteristics, and improving thermal stabilities and film-forming abilities to maximize effectiveness and usability. Furthermore, expanding the exploration of non-fish marine sources, such as invertebrates and algae, could provide alternative sustainable sources of collagen, contributing to a broader and more resilient supply chain. Despite these challenges, the remarkable growth of the marine collagen market reflects its potential to meet consumer demand for food and supplement industries.

## Figures and Tables

**Table 1:** Amino acid composition of collagen codfish skin, Atlantic salmon, sea bass scale, jellyfish (*R. esculentum*), and bovine commercial collagen (per 1000 residues).

Amino Acid	Bovine	Codfish (Cold Water)	Atlantic Salmon (Cold Water)	Sea Bass Scale (Warm Water)	Jellyfish (*R. esculentum*)
Alanine	102.04	91.48	115.87	133	108.6
Arginine	32.86	30.45	39	52	76.9
Aspartic acid	36.65	38.82	51.36	44	68.3
Cysteine	1.24	1.28	0.82	0	2.8
Glutamic acid	59.43	56.08	73.82	71	85.8
Glycine	296.44	266.12	344.01	327	267.9
Histidine	3.11	5.01	7.68	7	5.7
Isoleucine	6.74	5.61	10.31	11	30.5
Leucine	17.5	16.51	20.61	21	41.9
Lysine	22.2	19.62	24.79	27	51.0
Methionine	7.81	15.04	16.43	15	11.6
Phenylalanine	11.58	12.7	16.07	-	29.6
Proline	89.89	62.69	96.82	108	72.9
Serine	32.03	53.87	46.38	28	44.4
Threonine	13.2	16.89	23.18	24	36.5
Tyrosine	1.48	2.25	3	5	18.3
Valinine	12.86	12.02	16.56	22	38.0

Data presented are collected from previously published articles [19,21,26].

**Table 2:** Marine collagen source: classification, species, part, collagen type, and extraction method.

Classification	Name	Part	Collagen Type	Extraction Method	Extraction Yield (%)	References
	Alu-alu (*Sphyraena* sp.)	Skin	I	ASC	6.7	[28]
	Asian sea bass (*Lates calcarifer*)	Skin	I	ASC	59.31	[29]
	Bigeye tuna (*Thunnus obesus)*	Skin	I	ASC	13.5	[30]
	Bluefin tuna (*Thunnus orientalis*)	Skin	I	ASC	2.1	[31]
	Yellowfin tuna (*Thunnus albacares*)	Skin	I	ASC	61.26	[29]
Vertebrates	Round goby (*Neogobius melanostomus*)	Skin	I	ASC	10.0	[32]
	Silver catfish (*Pangasius* sp.)	Skin	I	ASC	4.2	[33]
	Lizardfish (*Saurida tumbil)*	Bone	I	ASC	-	[34]
	Blue shark (*Prionace glauca*)	Cartilage	II	PSC	-	[35]
	Parrotfish (*Scarus sordidus*)	Scale	I	ASC	1.17	[34]
	Amur sturgeon (*Acipenser schrenckii*)	Cartilage	I & II	ASC, PSC, SSC	27.04, 55.92, 2.18	[36]
	Grass carp (*Ctenopharyngodon idella*)	Swim bladder	I	PSC	38.98	[37]
	Catfish (*Silurus triostegus*)	Skin	I	ASC, PSC	23.92	[38]
	Sponge (*C. reniformis*)	Sponge tissue	IV	Enz	19.2	[39]
	Starfish (*Asterina pectinifera*)	Body wall	I	ASC	-	[40]
	Starfish (*Asterias pectinifera*)	Body wall	I	UAE	-	[41]
	Sea cucumber (*H. cinerascens*)	Body wall	I	ASC, PSC	72.2	[42]
	Jellyfish (*Rhopilema esculentum*)	Filament	I	PSC	4.31	[43]
Invertebrates	Jellyfish (*Stomolophus meleagris*)	Tissue	I	Enzyme	-	[44]
	Jellyfish (*Catostylus mosaicus*)	Tissue	I	ASC	-	[45]
	Byssus of Chilean mussels (*Mytilus chilensis*)	Mussels	I	ASC	3.86–7.56	[46]
	Mantis shrimp (*Miyakella nepa*)	Mussels	I	PSC	0.47	[47]

ASC: acid-solubilized collagen, PSC: pepsin-solubilized collagen, SSC: salt-solubilized collagen, and UAE: ultrasound-assisted extraction.

**Table 3:** Comparison of marine collagen extraction methods based on efficiency, cost, energy requirement, and impact on collagen structure.

Extraction Method	Efficiency	Cost	Energy Requirement	Impact on Collagen Structure
Salt-Solubilization Extraction (SSE)	Low to moderate; often combined with other methods	Low	Low	Minimal when optimized; low yield
Acid Extraction	Moderate to high; depends on acid type and concentration	Moderate	Low	Can reduce molar mass; may enhance gel strength
Alkaline Extraction	Moderate; effective for pretreatment of rigid materials	Moderate	Low to moderate	Risk of degradation of specific amino acids at high temp/concentration
Enzymatic Extraction	High; specific and controlled hydrolysis	High (enzyme cost)	Low	Preserves native structure; minimal degradation
Ultrasound-Assisted Extraction (UAE)	High; enhances mass transfer and extraction yield	Moderate to high (equipment cost)	Moderate	Preserves triple helix with controlled parameters
Microwave-Assisted Extraction	High; enhances cell matrix breakdown when combined with enzymes	High (equipment and energy cost)	High	May degrade structure if overheating occurs

**Table 4:** Food applications of marine collagen.

Collagen Source	Extraction Part	Collagen Forms	Function	Food Products	References
Fish processing byproducts (Tilapia)	Skin and bones	Peptides	Emulsifier	Butter and chocolate sauce	[152]
Fish collagen (commercial)	Skin and bone	Hydrolyzed collagen	Functional ingredient	Fruit juice	[153]
Fish collagen (Commercial)	-	Hydrolyzed collagen	Fat replacer	Buffalo patties	[154]
Seabass	Skin	Hydrolyzed collagen	Antioxidant	Herbal soup	[155]
Tilapia	Skin	Gelatin	Mechanical and water barrier	Food packaging	[156]
Jellyfish	Oral arm and umbrella	Gelatin	Ingredient	Cornmeal	[134]

## Data Availability

No data were collected for the content discussed in this article.
